# *In vitro *anti-angiogenic properties of LGD1069, a selective retinoid X-receptor agonist through down-regulating Runx2 expression on Human endothelial cells

**DOI:** 10.1186/1471-2407-11-227

**Published:** 2011-06-07

**Authors:** Jianjiang Fu, Wei Wang, Yu-Hui Liu, Hong Lu, Yongming Luo

**Affiliations:** 1 Department of Pharmacology, College of Pharmacy, Jiangxi University of Traditional Chinese Medicine, Nanchang, 330004 China; 2 Modern Education Technology Center, Jiangxi University of Traditional Chinese Medicine, Nanchang, 330004 China

**Keywords:** LGD1069, retinoid X receptor, metastasis, angiogenesis, Runx2

## Abstract

**Background:**

LGD1069 (Targretin^®^) is a selective retinoid X receptor (RXR) ligand, which is used in patients for cutaneous T-cell lymphoma. Our published study reported that LGD1069 inhibited tumor-induced angiogenesis in non-small cell lung cancer. In present study, we found that LGD1069 suppressed the proliferation, adhesion, invasion and migration of endothelial cells directly, and affected the expression of vegf and some matrix genes.

**Methods:**

Human umbilical vein endothelial cells (HUVECs) were used for *in vitro *study. MTT assay and Sulforhodamine B assay were used for cell viability assay; the tube formation assay was used to investigate the effect of LGD1069 on angiogenesis *in vitro*. *In vitro *adhesion, migration and invasion of HUVEC cells were analyzed by Matrigel adhesion, migration and invasion assay. Gene expressions were measured by RT-PCR and Western blot analysis.

**Results:**

Our data showed here that LGD1069 inhibited the activation of TGF-β/Smad pathway significantly. Furthermore, it was demonstrated that expression of Runx2 was suppressed pronouncedly during incubation with LGD1069. Runx2 is a DNA-binding transcription factor which plays a master role in tumor-induced angiogenesis and cancer cells metastasis by interaction with the TGF-β/Smad pathway of transcriptional modulators.

**Conclusions:**

Our results suggested that LGD1069 may impair angiogenic and metastatic potential induced by tumor cells through suppressing expression of Runx2 directly on human endothelial cells, which may point out new pathway through which LGD1069 display anti-angiogenic properties, and provide new molecular evidence to support LGD1069 as a potent anti-metastatic agent in cancer therapy.

## Background

Carcinomas are the most frequent type of human malignancies, and the vast majority of cancer deaths are caused by the formation of metastases rather than by the primary tumor itself [1]. Despite its clinical importance, spreading to distant organs remains the most insidious aspect of solid cancers, and the complexities about the genetic and biochemical determinants remain poorly understood. But for more than a century, cancer biologists paid lots of attention to this aspect. Based on current findings in this field, Gupta *et al*. posed a framework of cancer metastasis. There are several key steps in the biological cascade of metastasis: altered cellular adhesion and cell motility, disruption of the basement membrane and extracellular matrix, entry and survival in circulation, exit into new tissues and eventual colonization of a distant site [2]. In addition, the development of new vessels, or angiogenesis, contributes to the progressive growth and metastasis of solid tumors [3,4], and has already become an important target of anti-cancer therapy [5].

LGD1069 (Targretin) is a selective retinoid X receptor (RXR) ligand, termed rexinoid [6], which is used in patients for cutaneous T-cell lymphoma [7]. Several observations demonstrated that LGD1069 prevented and overcome acquired drug resistance in advanced breast cancer [8] and non-small cell lung cancer [9], and also in advanced prostate cancer [10]. Besides the efficacy on cancer therapy, LGD1069, as well as other retinoids such as *all-trans *retinoic acid, 9-*cis *retinoic acid and CD2409 etc., are frequently used for the treatment of skin disorders such as psoriasis, delayed-type skin hypersensitivity reactions, bullous diseases, and Kaposi's sarcoma, which are characterized by abnormal neovascularization [11].

Led by these clinical properties of LGD1069, we tested whether this rexinoid would cause similar effects on tumor neovascularization to those skin disorders previously. It was shown that LGD1069 inhibited tumor induced-angiogenesis through decreasing VEGF secretion via suppressing phosphorylation of JNK and ERK in non-small cell lung cancer [12]. So, we wondered whether there are direct impacts of LGD1069 on human endothelial cells. In the current study, it was reported that LGD1069 inhibited the proliferation, invasion and migration of endothelial cells directly, and affected the expression of vegf and matrix-related genes. Furthermore, we showed here that LGD1069 decreased the expression of Runx2, a master transcriptional factor which regulates angiogenesis and metastasis through specific DNA-binding motif, and the activity of TGF-β/Smad pathway significantly.

## Methods

### Materials

The RXR-selective retinoid used in this study, LGD1069, (Targretin^®^), was purchased from Sigma Chemicals (St Louis, MO). A stock solution (1 mM) was prepared by dissolving LGD1069 in DMSO and stored at 4°C for <1 month before use. The vehicle (DMSO) was used as a control in all experiments at a final concentration of 0.1%.

### Cell culture

HUVECs (Human umbilical vein endothelial cells) were isolated as previously described by Jaffe et al [13], from umbilical cords obtained from a parturient at Beijing Obstetric Hospital who gave written informed consent. The study protocol was approved by the IRB (Institutional Review Board) of Beijing Obstetric Hospital. HUVECs were routinely grown in M199 (Gibco, Grand Island, NY) supplemented with 10% fetal bovine serum (FBS) (Gibco) and endothelial cell growth supplement (ECGS) (BD Biosciences, Bedford, MA) at 37°C and 5% CO_2_. HUVEC between P3 and P4 were used for all experiments.

### MTT assay

Cell viability was assayed using a MTT assay. Briefly, log phase cells were plated in 96-well plates at a density of 3 × 10^4 ^cells per well in the presence of 2 ng/ml VEGF (physiological concentration) in 10% FBS media by for 24 h incubation, and then sufficient volumes of stock solution of LGD1069 and DMSO were added to the culture medium, in order to obtain different treatment doses. Then the vehicle and a range of LGD1069 were co-incubated with cells for different time. Three duplicate wells were set up in each sample. At least three independent experiments were carried out. After treatment, cells were incubated with 3-(4, 5-dimethylthiazol-2-yl)-2, 5-diphenyltetrazolium bromide (MTT, Sigma Chemical Company) (final concentration 0.5 mg/ml) for 4 h at 37°C. The media was carefully removed from each well and 200 μl of DMSO was added. The plates were gently agitated until the color reaction was uniform and the OD570nm and OD450nm were determined using a microplate reader (WellScan MK3, Labsystems Dragon). The data were analyzed using a SlideWrite program to determine the IC50 value of LGD1069. Media only treated cells served as the indicator of 100% cell viability.

### Sulforhodamine B assay

The growth inhibition effect of LGD1069 on endothelial cells was also examined with the sulforhodamine B (SRB) assay. Briefly, HUVEC cells were seeded in 96-well plates (2,000 cells per well) and co-incubated with 2 ng/ml VEGF overnight. Then, triplicate wells were treated with various concentrations of LGD1069. Three duplicate wells were set up in each sample. At least three independent experiments were carried out. After 96 h, the culture medium was discarded, 10% (w/v) pre-cooled (4°C) trichloroacetic acid (100 μl) was added to each well, and the cells were fixed at 4°C in a refrigerator for 1 h. Each well was then stained with 100 μl 0.4% SRB (w/v) in 1% acetic acid (v/v) for 15 min. The plates were washed with 1% acetic acid (v/v) for removal of unbound dye, air dried, and then treated with 150 μl per well 10 mM Tris base (pH 10.5) to dissolve the bound dye. The absorbance in each well was read with a microplate reader (WellScan MK3, Labsystems Dragon) at 515 nm. The data were analyzed using a SlideWrite program to determine the IC50 value of LGD1069. Media only treated cells served as the indicator of 100% cell viability.

### Tube Formation Assay

The tube formation assay was used to investigate the effect of LGD1069 on angiogenesis *in vitro*. The methods were described previously [5]. In briefly, a 96-well plate was coated with 80 μl liquid Matrigel per well, which was allowed to solidify at 37°C for 45 min. HUVEC cells were seeded at a density of 3 × 10^4 ^cells per well in 100 μl complete culture medium containing different concentrations of LGD1069 or vehicle (control), then 100 μl serum-free M199 medium contained VEGF (final concentration is 2 ng/ml) was added. Plates were incubated for 24 h at 37°C and 5% CO_2 _(sufficient for formation of an intact network in the control group). Images were recorded by an inverted microscope (Olympus, IX70, Japan), and total vessel lengths were counted with ImagePro plus 5.0 image analysis software (Media Cybernetics, Inc, Silver Spring, MD).

### *In vitro *assays of HUVECs adhesion

96-well microtiter plates were pre-coated with 10 μl Matrigel (0.5 μg/ml) and incubated at 4°C overnight. Wells were blocked with 20 μl M199 containing 2% BSA for 1h at 37°C. The exponential phase HUVE cells were harvested, and re-suspended in serum-free M199 medium supplemented with 0.1% BSA. The cells (5 × 10^4^/well) were seeded in wells and co-incubated with 2 ng/ml VEGF at 37°C in a 0.5% CO_2 _atmosphere for 2h with or without LGD1069. The wells were washed thrice with PBS to remove unattached cells, then the attached cells were incubated with MTT and the absorbance was measured at 570 and 450 nm. Each assay was performed in triplicate. Three independent experiments were repeated [14].

### Matrigel Invasion Assay

*In vitro *invasion of HUVEC cells was measured by the invasion of cells through 48-well microchemotaxis plates (AP 48, Neuro Probe, Gaithersburg, MD) with wells separated by a Polyvinylpyrrolidone-free polycarbonate filter with an 8 μm pore size [15]. Briefly, the polycarbonate membrane was pre-coated with 5 μg of fibronectin in a volume of 50 μl on the rough (lower) surface. The Matrigel was diluted to 100 μg/ml with cold PBS and applied to the smooth (upper) surface of the filters (5 μg/filter), and dried at room temperature. The lower compartments of the plates were filled with 30 μl M199 containing 0.1% BSA. Log-phase cells were harvested and washed thrice with serum-free M199, and re-suspended to a final concentration of 2 × 10^6^/ml in M199 with 0.1% BSA. Cell suspensions (100 μl) with or without LGD1069 were added to the upper compartment and with 2 ng/ml VEGF(final concentration) for 24 h at 37°C in a 5% CO_2 _atmosphere. The filters were fixed with methanol and stained with Haematoxylin for 10 min, washing with distilled water, and then stained with Eosin Y for 30s. The cells on the upper surface of the filters were removed by wiping with cotton swabs. The cells invading the lower surface of the filter through Matrigel and filter were manually counted under a microscope at a magnification of × 200, and each assay was performed in triplicate.

### Matrigel Migration Assay

*In vitro *migration of HUVECs was measured by AP 48 chamber (Neuro Probe, Gaithersburg, MD), similar to *in vitro *invasion assay. Briefly, the underside of polycarbonate membrane was coated with 20 μg/ml fibronectin overnight at 4°C. 30 μl of M199 (with 10% FBS and 10 μg/ml Collagen I) was added to the lower chamber, and the chamber was covered by filter. HUVECs were trypsinized and washed by FBS-free M199, then 100 μl of cell suspensions (in FBS-free M199, containing 2 × 10^5 ^cells) with or without LGD1069 were added to the upper chambers and co-incubated with 2 ng/ml VEGF overnight at 37°C. Determination of migrated cells is the same as what was described in Matrigel Migration Assay.

### RT-PCR assay

Untreated and treated cells with LGD1069 were washed twice with cold PBS. Total RNA was isolated by Trizol reagent according to the manufacturer's protocol (Invitrogen, Carlsbad, CA). RNA was dissolved in diethylpyrocarbonate (DEPC)-treated water (0.1% DEPC was added to water overnight and then autoclaved 20 min to destroy DEPC). According to the manufacturer's instructions (TaKaRa, Dalian, China), reverse transcription was performed at 42°C in the presence of 5 units of AMV reverse transcriptase and 1 μg of RNA for 60 minutes. AMV RT inactivation and RNA/cDNA/primer denaturation were performed at 95°C for 5 minutes. cDNA was stored at -20°C. The PCR profile was as follows, 10 min at 95°C, followed by several cycles of 30s at 95°C and 1 min at 60°C. The PCR product was separated by 2% agarose gel electrophoresis, and the gels were viewed by UV transillumination, photographed by Kodak 120 gel imaging system. Primers, Tm, and cycles were listed on Table 1.

**Table 1:** PCR Primers

	Primers	Product length(bp)	Tm (°C)
MMP-2	S:5'-GTGCTGAAGGACACACTAAAGAAGA	580	53
	A:5'-TTGCCATCCTTCTCAAAGTTGTAGG		

MMP-9	S:5'-CGGCACGGCAATGCTGAT	507	55
	A: 5'-AGGGCGAGGACCATAGAGG		

VEGF	S:5'-TCCAGGAGTACCCTGATGAG	204	49
	A:5'-ATTCACATTTGTGTGCTGT		

β-actin	S:5'-GTGGGGCGCCCCAGGCACCA	540	55
	A:5'-CTTCCTTAATGTCAVGCACGATTTC		

### MMPs activities assay

MMPs activities were measured by SensoLyte^® ^520 MMPs Assay Kit according to the manufacturer's protocol (AnaSpec, Fremont, CA). Briefly, Untreated and treated cells with LGD1069 were collected and homogenized by assay buffer containing 0.1% Triton-X 100, and centrifuged for 15 min at 10000X g at 4°C. Collect the supernatants and store at -70°C until use. To activate MMPs before the measurement, the supernatants were incubated with 4-aminophenylmercuric acetate (APMA) for 1 h at 37°C. Then, 50 μl MMPs-containing samples were added to 96-well plate per well. Add 50 μl per well of MMPs substrate solution to the sample and control wells. Mix the reagents by shaking the plate gently for 30 sec. Incubate the reaction at 37°C for 50 min. 50 μl stop solution was added per well, and mix the reagents and measure fluorescence intensity at Ex/Em = 490/520 nm(Victor3, Perkin Elmer, Waltham, MA).

### Western blot analysis

HUVECs were washed once with PBS and then lysed by the addition of 1 ml lysis buffer (10 mmol/l Tris, pH 7.6, 150 mmol/l NaCl, 5 mmol/l EDTA, pH 8.0, 10 ml/l Triton X-100, 1 mmol/l DTT) containing 0.1 mmol/l PMSF. After 30 min on ice, lysates were collected and clarified by centrifugation at 15,000 g for 10 min at 4°C. Aliquots of whole cell lysates were subjected to 10% SDS-PAGE and then transferred to Hybond nitro blotting membranes. The membranes were blocked with 3% bovine serum albumin in Tris-buffered saline containing 0.5 ml/l Tween-20 (TTBS) and then probed with either anti-Smad2/3(sc-6032), p-Smad2/3(sc-11769), Runx2(sc-8566), and anti-β-actin (sc-1616) antibodies in 30 g/l bovine serum albumin in TTBS. Positive antibody reactions were visualized using UVP EC3-410 luminescence system.

### Statistical analysis

The mean values were obtained from at least three independent tests. The data are presented as mean ± S.D., and analyzed with the SPSS software program (version 10.0 for Windows; SPSS INC., Chicago, IL). Comparison among different groups was carried out by analysis of variance (the one-way ANOVA). Differences between means were considered statistically significant at p < 0.05.

## Results

### Inhibition of proliferation on endothelial cells

**Figure 1:**
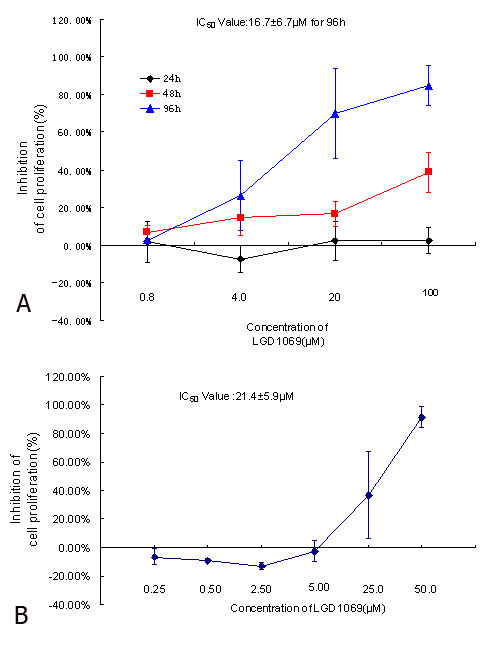
**Anti-proliferative properties of LGD1069 in endothelial cells**. Panel A showed the results analyzed by MTT assay. Cells were incubated with LGD1069 in the presence of 2 ng/ml VEGF, at different concentrations for 24, 48 and 96h, respectively. The IC_50 _value for 96h was 16.6 ± 6.7 μM. Panel B is the results of SRB assay. The HUVEC cells were treated with LGD1069 for 96h at different concentrations, and the IC_50 _value was 21.4 ± 5.9 μM. Vehicle (DMSO) was used as a control in both of the viability assay. Data were expressed as mean ± SD. (n = 3).

To examine whether LGD1069 was capable of inhibition the proliferation of HUVEC, we performed MTT assay with a range of LGD1069 and different time. HUVECs were treated with 0.8, 4, 20 and 100 μM LGD1069 for 24h, 48h and 96h, respectively. The inhibition of LGD1069 on HUVECs was given in Figure 1A. Data showed that treatment of LGD1069 for 96h resulted in a dose-dependent inhibition of cells proliferation with the IC_50 _value of 16.6 ± 6.7 μM when the cells were co-incubated with VEGF at 2 ng/ml. A slight inhibition was observed on the cells incubated with LGD1069 for 48h. But on the cells treated for 24h, there were no inhibition of proliferation. In addition, we also tested the effects of LGD1069 on HUVECs viability by SRB assay. It's shown that treatment with LGD1069 for 96h resulted in growth inhibition, similar to the results of MTT assay. The IC_50 _value was 21.4 ± 5.9 μM (Figure 1B).

From these results, we concluded that alteration of HUVECs viability depended on the final concentration and incubated time of LGD1069. Therefore, the investigations of antiangiogenic effects on HUVECs would be carried out at proper concentrations and exposure time, which did not cause significant proliferative inhibition.

### LGD1069 directly decreased HUVECs tube formation

**Figure 2:**
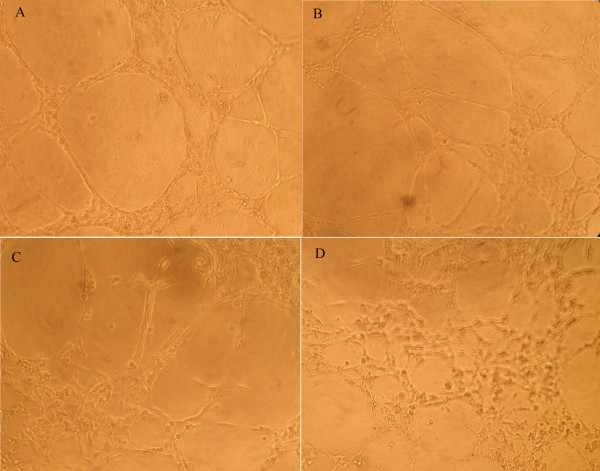
**Effects of LGD1069 on tube formation of HUVECs induced by VEGF**. HUVECs were seeded in Matrigel-coated 96-well plates, and untreated or treated with LGD1069, in the presence of 2 ng/ml VEGF. Vehicle (DMSO) was utilized as a control. A. Control; B. LGD1069 7.5 μM; C. LGD1069 15 μM; D. LGD1069 30 μM (100X).

Capillary formation starts with endothelial cell differentiation and tube formation *in vitro *is a consequence of endothelial cell differentiation. We tested whether LGD1069 decreased the formation of tubes by HUVECs on Matrigel induced by VEGF *in vitro*. Control HUVECs formed a mesh of tubes within 24 h (Figure 2 and Table 2), whereas those treated with LGD1069 did not. HUVECs treated with low concentrations (7.5 and 15 μM) of LGD1069 differentiated into short tubes but were unable to form meshes, whereas those treated with higher concentrations (30 μM) remained dotted on the Matrigel without obvious morphological changes. As shown in Figure 1a, treatment of HUVECs with 100 μM for 24h showed 2.27% inhibition of cell growth rate, indicating that the observed ability of LGD1069 to inhibit tube formation was not due to cell growth inhibition.

**Table 2:** Microvessel Formation of endothelial Cells *in vitro *(24h)

Group	Total Vessel Actual Perimeter Length (APLT) ± SD(μm)	Formation Rate (%)
CTRL	835.2 ± 91.1	100.00%
LGD1069(7.5 μM)	761.9 ± 59.7	91.22%
LGD1069(15 μM)	601.8 ± 37.4^a^	72.05%
LGD1069(30 μM)	109.2 ± 10.6^b^	13.07%

HUVECs were seeded in Matrigel-coated 96-well plates, and untreated or treated with LGD1069, in the presence of 2 ng/ml VEGF. Vehicle (DMSO) was utilized as a control. Five fields were counted for each well, and each concentration repeated three wells. Data were expressed as mean ± SD. a p < 0.05, b p < 0.01 compared with control (n = 3).

### LGD1069 inhibited HUVECs invasion through reconsititutive basement membrane

**Figure 3:**
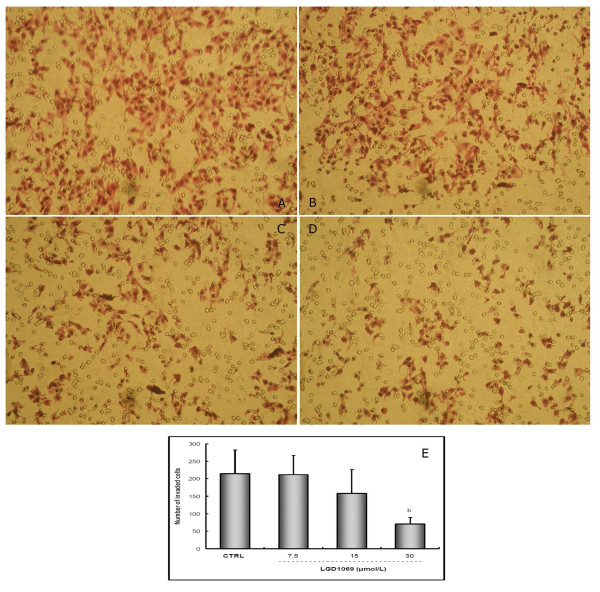
**LGD1069 inhibited the invasion of HUVECs in AP48 chamber**. HUVECs were seeded in upper compartments of AP48 chamber, and untreated or treated with LGD1069, in the presence of 2 ng/ml VEGF. Cells on the lower face of the membrane were counted by the method described under materials and methods. Vehicle (DMSO) was utilized as a control. Five fields were counted for each chamber, and each concentration repeated three chambers (100X). A. Control; B. LGD1069 7.5 μM; C. LGD1069 15 μM; D. LGD1069 30 μM; E. statistical results. b p < 0.01 compared with control (n = 3).

As endothelial cells invasion is critical for the processes of angiogenesis and metastasis, we studied the effect of LGD1069 on HUVECs invasion *in vitro*. In this study, we used fibronectin as a chemo-attractant on the lower surface of polycarbonate membrane. Here, we found that HUVECs invasion was dose-dependently blocked by LGD1069 (Figure 3), with inhibition of 1.31, 26.40 and 66.88% at concentrations of 7.5, 15 and 30 μM LGD1069 in normal culture conditions, respectively.

### LGD1069 suppressed HUVECs migration

**Figure 4:**
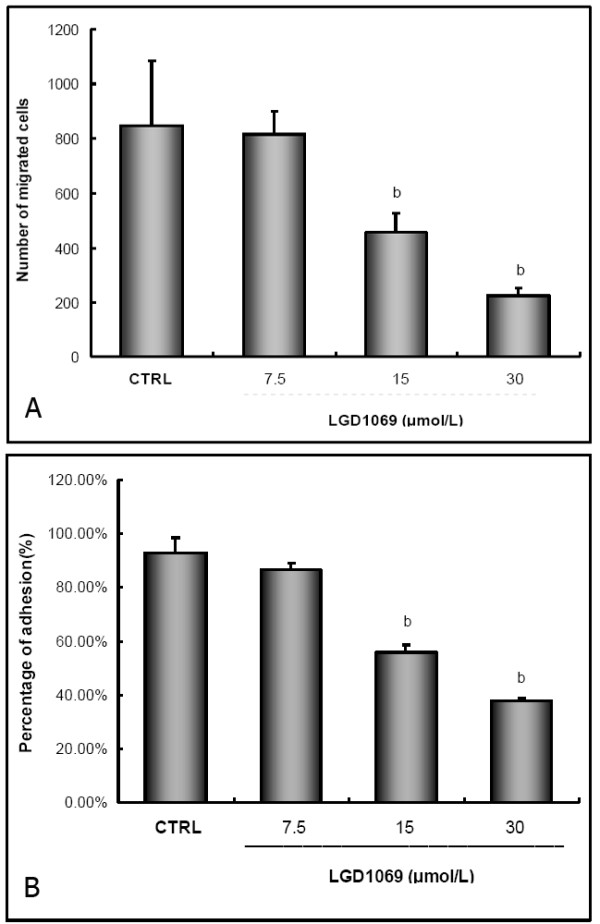
**LGD1069 inhibited the migration and adhesion of HUVECs**. For migration assay, HUVECs were seeded in upper compartments of AP48 chamber, and untreated or treated with LGD1069, in the presence of 2 ng/ml VEGF. Cells on the lower face of the membrane were counted by the method described under materials and methods. Vehicle (DMSO) was utilized as a control. b p < 0.01 compared with control (n = 3). For adhesion assay, HUVECs were seeded in Matrigel-coated 96-well plates, and untreated or treated with LGD1069, in the presence of 2 ng/ml VEGF. The number of adhesive cells was evaluated by MTT assay. Vehicle (DMSO) was utilized as a control. Values were normalized to the mean number of adhesive control cells and presented as mean ± SD percentage of adhesion (n = 3). b p < 0.01 compared with control. Panel A is results from migration assay, and panel B shows the results of adhesion assay.

With similar methods to invasion assay, effects of LGD1069 on HUVEC migration *in vitro *were tested. In this study, we used fibronectin as a chemo-attractant on the lower surface of polycarbonate membrane, but no matrigel was used. Here, we found that migration of HUVECs was dose-dependently blocked by LGD1069 (Figure 4a), with inhibition of 3.80, 46.13 and 73.75% at concentrations of 7.5, 15 and 30 μM LGD1069 in normal culture conditions, respectively.

### LGD1069 decreased HUVEC adhesion *in vitro*

Since adhesion of endothelial cells to the basement membranes is an important step of angiogenesis and metastasis, we also examined the effects of LGD1069 on HUVECs adhesion *in vitro *when the cells were co-incubated with VEGF at 2 ng/ml. As shown in Figure 4b, the adhesion rate to Matrigel was 86.49, 55.73 and 38.01% when HUVECs were incubated with 7.5, 15 and 30 μM LGD1069 for 2 hours. It's revealed that LGD1069 could inhibit endothelial cell adhesion to the component of basement membrane in a concentration-dependent fashion.

### LGD1069 suppressed MMPs activation

**Figure 5:**
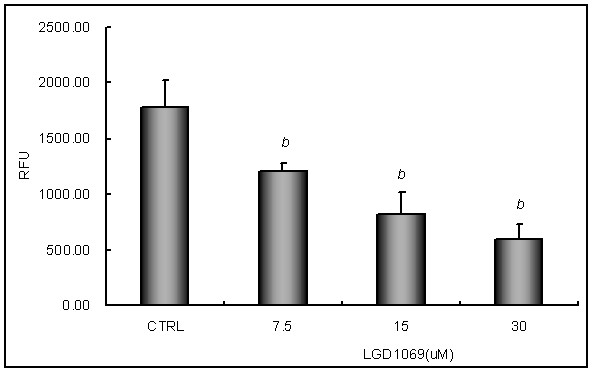
**Effects of LGD1069 on metalloproteinases activities in HUVECs**. HUVECs were incubated for 12 h in medium containing vehicle (DMSO, Line 1) or 7.5 μM, 15 μM, and 30 μM LGD1069, in the presence of 2 ng/ml VEGF. Data were expressed as mean ± S.D. a p < 0.05, b p < 0.01 compared with control.

To determine whether LGD1069 influenced the activities of endothelial cells-secreted- metalloproteinases to inhibit invasion and angiogenesis, we measured the MMPs activities in HUVECs by FRET-based assay. It's revealed that LGD1069 suppressed FRET substrate cleavage significantly in a does-dependent manner (Figure 5).

### LGD1069 impacted expression of matrix genes

**Figure 6:**
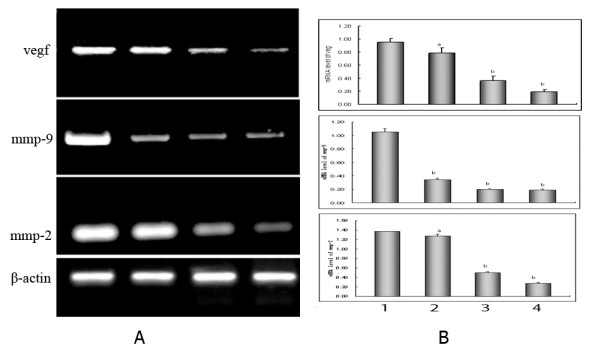
**Effects of LGD1069 on metalloproteinases and vegf mRNA expression in HUVECs by RT-PCR assay**. HUVECs were incubated for 12 h in medium containing vehicle(DMSO, Line 1) or 7.5 μM (Line 2), 15 μM (Line 3), and 30 μM (Line 4) LGD1069, in the presence of 2 ng/ml VEGF. The expression of a housekeeping gene β-actin was used to normalize gene expression level. Error bars represent the standard deviation of three replicate experiments. Panel A: RT-PCR analysis of metalloproteinases and vegf genes expression. Line M was DNA marker. Pane B: densitometric analysis of electrophoretic bands. Intensities were normalized to a β-actin signal. Data were expressed as mean ± S.D. a p < 0.05, b p < 0.01 compared with control.

Then, expression of matrix metalloproteinases (mmps) in HUVECs were examined by RT-PCR assay, in order to find out whether LGD1069 down-expressed endothelial cell-secreted metalloproteinases to decrease its activities. It's revealed that LGD1069 decreased the expression of mmp-2 and mmp-9 at RNA level (Figure 6a and 6b).

### LGD1069 down-regulated activation of Smad2/3 and expression of Runx2

**Figure 7:**
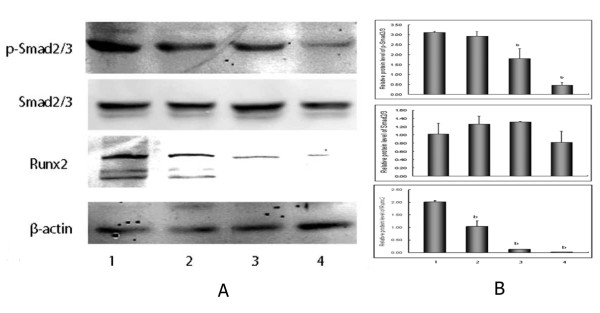
**Suppression of p-Smad2/3 phosphorylation and Runx2 expression in human endothelial cells after incubation with LGD1069 for 24 h, in the presence of 2 ng/ml VEGF**. (A) Western blot analysis of protein lysates. (B) densitometric analysis of electrophoretic bands. Intensities were normalized to a β-actin signal. Line 1: Control; Line 2: LGD1069 7.5 μM; Line 3: LGD1069 15 μM; Line 4: LGD1069 30 μM. Vehicle (DMSO) was utilized as a control. Data were expressed as mean ± SD; b p < 0.01 compared with control (n = 3).

We also tested the effects of LGD1069 on expression level of Runx2 in HUVECs when VEGF at pathologic level was present. Western Blotting assay demonstrated that LGD1069 decreased the expression of Runx2 at protein levels (Figure 7a and 7b). It's revealed that LGD1069 significantly suppressed Runx2 expression in a dose-dependent fashion. Since Runx2 is a DNA-binding transcription factor that interacts with the TGFβ/Smad family of transcriptional modulators to stimulate cell proliferation and tumor progression, we looked for the signs of LGD1069 affecting the phosphorylation of Smads in HUVECs simulated by VEGF. Western blotting showed that LGD1069 treatment down-regulated the phosphorylation of Smad2/3 significantly, but there are no changes of the expression of total Smad2/3.

## Discussion

LGD1069, a selective retinoid X receptor agonist, is used in patients for cutaneous T-cell lymphoma [7], and is also demonstrated that it is an efficacious chemopreventive and chemotherapeutic agent in a number of preclinical rodent models breast cancer [7,16-18]. Furthermore, LGD1069 was shown recently to prevent tumor-induced angiogenesis and metastasis. Yen and his colleagues determined the influence of this retinoid X receptor agonist on angiogenesis and metastasis in solid tumors [19]. Moreover, in previous study, we also demonstrated that LGD1069 decreased tumor-induced angiogenesis. In that study we found LGD1069 impair the capacity of vessel formation induced by tumor cells via suppression of VEGF secretion in human non-small cell cancers. Thus, we sought to determine whether LGD1069 exerts its anti-angiogenic ability by directly impairing activation of human endothelial cells and to identify the underlying story here. In the current study, we found that LGD1069 inhibited proliferation and survival of endothelial cells, and destroyed vessel-network formation on Matrigel. In addition, *in vitro *analysis indicated that LGD1069 attenuated endothelial cell adhesion, migration and inhibited cell invasion through reconstituted extracellular matrix (ECM).

Angiogenesis denotes the formation of new blood vessels from pre-existing vessels. Angiogenetic factors, such as fibroblast growth factors (FGFs) and vascular endothelial growth factors (VEGFs), regulate endothelial cells to secrete several proteases (such as MMPs and TIMPs) and plasminogen activators (such as uPA), resulting in the degradation of the vessel basement membrane, and the formation of stable new vessels[20-22]. Besides activating endothelial cells, VEGFs binding to their specific receptors expressed on cell surface, also regulate proliferation and survival of endothelial cells, and microvascular permeability [19; 23]. Led by these findings and our published study, we tested whether there are effects on biological behaviors of ECM related enzymes. Our data showed that at the presence of VEGF (2 ng/ml), LGD1069 inhibited expression of MMP-2, MMP-9 in endothelial cells. Collectively, these results indicated that the anti-angiogenetic and anti-metastatic properties of LGD1069 may be due to the inhibition of expression of matrix-related proteases in endothelial cells, resulting in inhibiting endothelial cell adhesion, migration, invasion and proliferation. These data suggest that LGD1069 may impede the activation of endothelial cells directly, and its effects on angiogenesis and metastasis may occur through activation of protease expression and events that regulate angiogenesis.

Runx2 is a member of the runt family of DNA-binding transcription factor which possesses a highly conserved DNA binding and protein-protein interaction domain. It plays essential roles for the formation of skeleton[24]. Qiao *et al*. reported that Runx2 DNA-binding activity is elevated in proliferating endothelial cells [25]. Recent Studies showed that Runx2 mediates endothelial cell migration and invasion during tumor angiogenesis [26]. Furthermore, it was found that Runx2 is ectopically expressed in metastatic cancer cells but not in non-metastatic cancer cells, and that several genes required for bone development and turnover, such as opn, bsp, mmp12 etc., are activated by Runx2 [27]. It' well known that this cohort regulated by Runx2 is essential mediators of tumor invasion and metastasis. Pratap *et al*. also demonstrated that Runx2 activates the "vicious cycle" of TGF- β-mediated tumor growth and metastatic bone disease [26]. Therefore, Harada and Rodan *et al*. presumed that Runx2 is a "master" transcription factor in the development of metastatic foci in several solid tumor [28]. Given the important role of Runx2 in cancer-induced angiogenesis and metastasis, we wondered whether it plays a role in the inhibition of MMP-2 and MMP-9 expression induced by LGD1069. The following specific studies will test this hypothesis. Data from current study suggested that LGD1069 inhibited expression of Runx2 and phosphorylation of Smad2/3, but no effects were found on expression of total Smad2/3.

In conclusion, we demonstrated here that LGD1069 may impairs metastatic potential in solid tumor, and the inhibitory effects of LGD1069 were due to its ability to inhibit endothelial cell growth and interfere with adhesion, migration and invasion of endothelial cells directly. These effects of LGD1069 on endothelial cells may be associated with inhibition of activation of Runx2 protein by interacting with the TGF-β/Smad family. These findings have important implications for patients with malignant diseases. For example, LGD1069 can be used alone or incorporated with other conventional modalities such as radiation and chemotherapy to improve the efficacy of these treatments as well as to prevent development of distant metastasis.

However, there are several questions arising from our data. Such as, whether is there a direct interaction between Runx2 and RXRs, which both are DNA-binding transcription factors? Whether are the effects on cell behaviors and expression of molecules of LGD1069 coincidental or directly causes and effects? Recently, the work from Hoover *et al*.[29] may provide a little bit clues, but not all. They reported that retinoids, such as LGD1069 and *9-cis *RA, directly regulated Smad activation and sub-cellular accumulation via RXR-α[29]. From these data, it seems that there exist indirect association between RXRs and Runx2. Therefore, it is worthwhile to further explore the role and regulation in of Runx2 in endothelial cells, and to figure out the cross-talk between Retinoic receptors and Runx2 activity. On-going research is directed towards understanding the underlying story of effects of LGD1069 on Runx2 in endothelial cells and tumor cells. Information obtained from these studies will have significant impact on the therapeutic use of LGD1069 in treatment of those malignant diseases.

## Conclusions

Our results suggested that LGD1069 may impair angiogenic and metastatic potential induced by tumor cells through suppressing expression of Runx2 directly on human endothelial cells, which may point out new pathway through which LGD1069 display anti-angiogenic properties, and provide new molecular evidence to support LGD1069 as a potent anti-metastatic agent in cancer therapy.

## Competing interests

The authors declare that they have no competing interests.

## Authors' contributions

JF took responsibility for the design of this project, analysis and interpretation of data. HL contributed to HUVECs culture. YHL carried out MTT, SRB assays and adhesion assay. WW was responsible for PCR and Western blotting. YL participated tube formation assay, Matrigel invasion assay and migration assay, and wrote the manuscript. All authors had read, edited and approved the final manuscript.

## Pre-publication history

The pre-publication history for this paper can be accessed here:

http://www.biomedcentral.com/1471-2407/11/227/prepub
